# Development and Evaluation of an Online Fall-Risk Questionnaire for Nonfrail Community-Dwelling Elderly Persons: A Pilot Study

**DOI:** 10.1155/2016/1520932

**Published:** 2016-05-10

**Authors:** Seraina Obrist, Slavko Rogan, Roger Hilfiker

**Affiliations:** ^1^School of Health Sciences, University of Applied Sciences and Arts Western Switzerland Valais, Rathausstrasse 8, 3954 Leukerbad, Switzerland; ^2^Department of Health, Discipline of Physiotherapy, Bern University of Applied Sciences, Murtenstrasse 10, 3008 Bern, Switzerland

## Abstract

*Introduction*. Falls are frequent in older adults and may have serious consequences but awareness of fall-risk is often low. A questionnaire might raise awareness of fall-risk; therefore we set out to construct and test such a questionnaire.* Methods*. Fall-risk factors and their odds ratios were extracted from meta-analyses and a questionnaire was devised to cover these risk factors. A formula to estimate the probability of future falls was set up using the extracted odds ratios. The understandability of the questionnaire and discrimination and calibration of the prediction formula were tested in a cohort study with a six-month follow-up. Community-dwelling persons over 60 years were recruited by an e-mail snowball-sampling method.* Results and Discussion*. We included 134 persons. Response rates for the monthly fall-related follow-up varied between the months and ranged from low 38% to high 90%. The proportion of present risk factors was low. Twenty-five participants reported falls. Discrimination was moderate (AUC: 0.67, 95% CI 0.54 to 0.81). The understandability, with the exception of five questions, was good. The wording of the questions needs to be improved and measures to increase the monthly response rates are needed before test-retest reliability and final predictive value can be assessed.

## 1. Introduction

Falls are a common cause of accidents and they can have serious consequences ranging from fear of falls to fractures, loss of independency, or even mortality. Approximately 25% of people over 65 years of age and living at home fall each year and about 20% of the falls require medical attention [1]. Mortality after a falls-related hospitalisation is high [2] and the falls specific mortality is still rising, although the mortality due to fractures after falls is declining [3]. Forty percent of the admissions to a long-term stay in a nursing home are due to a fall. Therefore, prevention of falls or their consequences is important. There exist a plethora of known risk factors for falls [4, 5] and the risk factors generally increase with age. However, older people are often not aware of their own fall-risk [6]. They are aware of the increased fall-risk of other elderly persons, but they are often convinced that this does not apply for themselves [7]. Furthermore, some older adults are reluctant to admit that they are at risk for falls because they fear that their families might send them to nursing homes [8, 9]. Health professionals, such as nurses or physiotherapists, might play an important role in raising the awareness of the fall-risk.

Screening for falls is usually performed by a health professional. However, in the group of the “young old,” not all are regularly seeing health professionals, or they are seeking care for other health conditions and the potentially increased fall-risk is not recognised or not perceived as an issue and not targeted by them or the health professionals [10]. A self-assessment tool might increase the awareness of the fall-risk and the motivation to discuss the problem with a health professional and to start a preventive programme [11, 12].

Current self-administered predictions tools do not cover all dimensions of fall-risk, such as dual tasks, medication, diseases like diabetes, pain, stroke, rheumatic disease, fear of falling, the frequency of toileting, gait problems, balance, muscle weakness, sensibility impairments, or hearing problems [13–17] (see also Table 1 for a comprehensive overview of existing tools).

Therefore, based on a search for systematic reviews and meta-analysis on risk factors for falls, we set out to (a) collect risk factors that were consistently reported in studies, (b) to extract coefficients from predictive models, (c) to devise a comprehensive set of questions, and (d) to test, in a sample of community-dwelling persons aged sixty years or older, the feasibility, understandability, calibration, and discrimination using the extracted coefficients, including the continuous assessment of falls during a six-month follow-up period. We hypothesize that (a) the monthly response rate is higher than 80%, (b) that the understandability of the questions is good, (c) that the self-predicted fall-risk is not in agreement with the observed fall-risk, (d) that the observed fall-risk is associated with the predicted fall-risk, and (e) that we can discriminate between fallers and nonfallers based on the risk score calculated with the coefficients from the literature and our self-reported questionnaire.

## 2. Materials and Methods 

This study included several steps: (1) defining a set of predictors for falls based on published meta-analyses, (2) devising a set of questions for the self-assessment of the risk factors out of seven questionnaires, and (3) prospective cohort study to assess the feasibility and the preliminary predictive values of the online assessment of the fall-risk.

### 2.1. Defining the Set of Predictors

We searched in PubMed for systematic reviews and meta-analyses on risk factors in community-dwelling elderly people; search strategy: (((risk OR odds OR predict^*∗*^ OR likelihood OR sensitivity OR specificity OR AUC OR ROC OR calibration OR discrimination))) AND ((((((falls [title]) OR fall [title]) OR faller [title])) AND ((meta-analysis [Publication Type]) OR systematic review [title])) AND ((elderly OR older OR aged OR senior OR seniors))). Inclusion criteria were systematic reviews and meta-analysis on prospective cohort studies including community-dwelling elderly persons. We extracted the risk factors for falls that were statistically significant in the meta-analyses. For each factor we extracted the coefficients (i.e., log of the odds ratio) for the prediction of falls in community-dwelling older adults from the meta-analysis with the most included participants or studies for the given predictor.

### 2.2. Devising the Set of Questions

Based on seven existing questionnaires for the self-assessment of fall-risk [13–17, 44, 45], we devised a set of questions that covered most of the fall-risks found in the previous step (review of reviews). The questions were written in German and translated into French and submitted to seven health professionals and two laypersons with the question about the understandability. Amendments were made if necessary.

We included ten questions about personal characteristics and a question about the self-perceived risk of falling, as well as the understandability (comprehension of the questionnaire) and suggestions for different formulations.

The questionnaire was implemented in an online survey system (SurveyMonkey [46]).

For the monthly follow-up we assessed whether a person fell during the last months and the number of falls. A fall is often defined as “an event which results in a person coming to rest inadvertently on the ground or floor or another lower level.” [47]. For our study, we decided to exclude falls in sports activities such as biking, skiing, or mountaineering. Based on feedback from participants at the first monthly follow-up, we added a question about the activity at which the falls occurred and two questions to assess the level of physical activity as recommended by Gill et al. [48] for the later follow-ups.

### 2.3. Prospective Cohort Study

The main part of this study was a longitudinal cohort study with a six-month follow-up (falls assessment and assessment of physical activity). Study participants were community-dwelling elderly persons aged 60 years or more. They had to be able to walk independently, with or without walking aids. German and French speaking participants were included if they had an e-mail address.

Participants were recruited by a snowball-sampling method [49]. This method allows the inclusion of participants that are difficult to achieve. If our hypothesis is true that our target population has a low awareness of their risk to fall, they would, for example, most probably not respond to other sampling methods such as information leaflets or advertisements in journals. Other sampling methods such as phone number lists are nowadays not valid anymore, because a large subset of the population is not listed in directories (phone books). A first set of e-mails with a link to the online survey (SurveyMonkey) was sent to acquaintances with a description of the target population (i.e., describing inclusion criteria); they were then asked to send the e-mail to their acquaintances, and so on. For six months, the monthly fall assessment was sent by e-mail via SurveyMonkey.

*Sample Size*. We used a convenience sample consisting of the 134 participants responding to the e-mails sent out with the snowball method. This sample size allowed the estimation of the incidence of falls and univariable association between risk factors and falls with enough statistical precision.

The project was conducted in accordance with the Declaration of Helsinki (1964) and was approved by the relevant ethical committee (CCVEM 014/14). All participants provided informed consent to the participation.

### 2.4. Adaptation of the Questionnaire

Based on the feedbacks on the understandability and the suggestions for alternative formulations, propositions for amendments were prepared. The final amendments will be part of a future project including a larger sample of experts including elderly persons.

### 2.5. Statistical Analysis

Descriptive statistics were presented as mean and standard deviation or as proportions, as appropriate. To express the association between risk factors and falls we calculated odds ratios and risk ratios and corresponding 95% confidence intervals. We used Stata Version 14.0 [50]. We calculated both risk and odds ratios because risk ratios are easier to interpret but the odds ratios allow a better comparison with published prediction tools. If a participant did not return a monthly falls follow-up, we assumed that there was no fall in this month.

To test the hypothesis that the participants are not aware of their fall-risk, that is, their self-perceived fall-risk is lower than the actual fall-risk, we calculated the proportion of fallers within each category of the self-perceived risk and calculated a chi-squared test with the null hypothesis that there is no association in the perceived fall-risk with increasing observed fall-risk.

### 2.6. Prediction Formula

Because our sample size was only large enough for univariable analyses and too low for the fitting of a robust multivariable prediction model, we used the coefficients published in the meta-analyses. The prediction formula consisted of a scoring function and a logistic probability function, where the scoring function reads as follows:

 scoring function = −4.5 + 0.1044 *∗* (age over 60/5) + 1.351 *∗* fallen last 12 months + 0.495 *∗* low spirit at some days + 0.548 *∗* incontinence + 0.62 *∗* need get up night + 0.215 *∗* rheumatic disease + 0.307 *∗* diziness + 0.779 *∗* neurological disease + 0.239 *∗* diabetes + 0.445 *∗* dichotomous pain + 0.247 *∗* high blood pressure + 0.47 *∗* heart symptoms + 0.875 *∗* fear of falls + 0.94 *∗* walk slower + 0.742 *∗* walking aids + 0.2852 *∗* perceived dual task problem + 0.859 *∗* self perceived balance + 0.457 *∗* any range of motion limitation lower extremity + 0.788 *∗* sensory deficit lower extremity + 0.399 *∗* vision problem + 0.315 *∗* do not hear good + 0.548 *∗* dichotomous home hazards + 0.718 *∗* low BMI + 0.8242 *∗* ADL need help + 0.637 *∗* fracture + 0.54 *∗* polymedication + 1.445 *∗* any medication + 0.24 *∗* postural hypotension + 0.98 *∗* difficult get up chair because of weak legs.

 And the logistic probability function is as follows: 1/(1 + exp(−1*∗*scoring  function)).

This formula has to be considered as preliminary because the coefficients of each predictor are not adjusted for all other predictors, which leads to an overestimation of the fall-risk. The coefficients need to be adjusted, for example, by the means of methods proposed by [51]. These methods need larger sample sizes than we had in our study.

Based on this preliminary prediction formula, we tested the calibration of the prediction model with a calibration plot (observed versus predicted falls) and a Hosmer-Lemeshow test. The discrimination (i.e., the ability to detect fallers) was tested with a receiver operating characteristic (ROC) curve and the area under the ROC-curve.

## 3. Results

The systematic search for systematic reviews and meta-analysis on fall-risk factors yielded 113 abstracts from which 14 systematic reviews were included [4, 5, 40, 42, 43, 52–60]. Because we extracted the coefficients from the meta-analysis with the most participants or studies included, the coefficients were taken from the newest reviews [4, 5, 40, 42, 43]. In addition, we extracted the coefficients from one single study for the variable frequent toileting [41] because we preferred this variable over the variables urinary incontinence or urinary functional sign published in the Block 2013 meta-analysis.  Table 2 shows the set of extracted factors as well as its odds ratio, coefficients, and heterogeneity, if available.

### 3.1. Set of Items Devised for the Self-Administered Fall-Risk Questionnaire

Based on the set of predictors we devised a set of questions. Because there was considerable overlap between the predictors, we selected a subset of 29 predictors with the aim of reducing overlap. Because some constructs were covered with more than one question, our questionnaire consisted of 36 questions, including demographic characteristics. Some of the questions consisted of several response options covering different risk factors.

### 3.2. Characteristics of Included Participants

**Figure 1:**
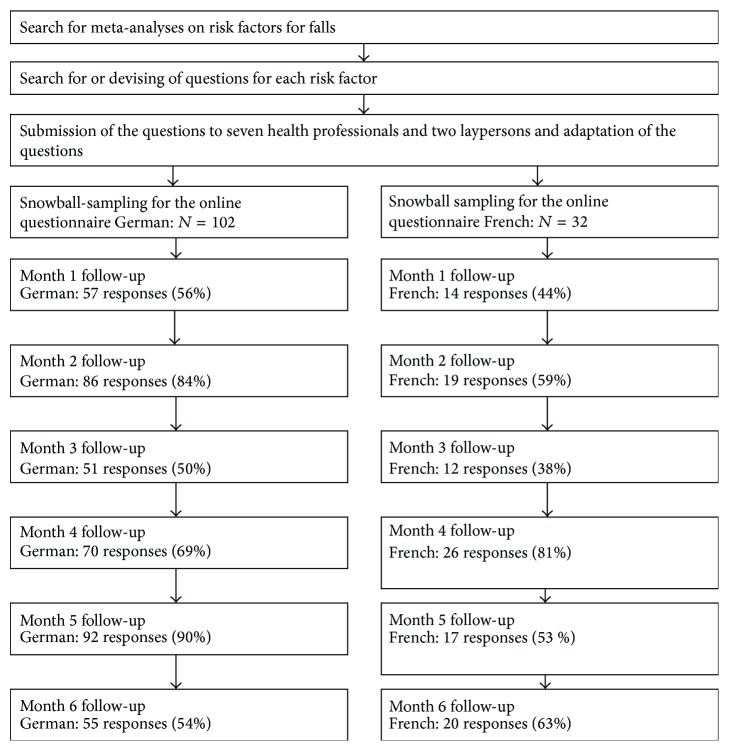
Study diagram.

With the snowball-sampling we could include 134 participants. The response rate during the monthly follow-up varied from 38 to 90% (see Figure 1). The mean age of the 134 participants was 69.3 years with a standard deviation of 5.6 years. There were slightly less women than men (45% women and 55% men). The mean body mass index (BMI) was 25.95; 13% had a BMI of 30 or more (i.e., would be classified as obese). The proportion of participants who did fall during the last twelve months was 18%; only a very small proportion had consequences due to these falls. During the 6-month follow-up, 32 participants did fall at least once, we excluded seven falls (three falls on bike, one fall on ski, two falls on icy roads, and one fall during mountaineering on steep paths), resulting in 25 falls (18.7%). For each risk factor, only a small proportion of participants indicated problems which leads to wide confidence intervals in the odds ratios (Table 2) and the risk ratios presented (Table 3).

### 3.3. Self-Perceived Fall-Risk and Actual Falls

For the question about the self-perceived probability to fall within the next six months, 49 participants (37%) reported that they “will not fall” and 7 (14% of the 49) did actually fall; 81 (60%) reported that they “will probably not fall” and 17 (21% of the 81) did fall. Only two persons reported that they will “probably fall” and one of those did fall. Two participants did not respond to the question about the self-perceived fall-risk. There was no association between self-perceived and observed fall-risk (*p* = 0.338).

### 3.4. Predictive Values

**Figure 2:**
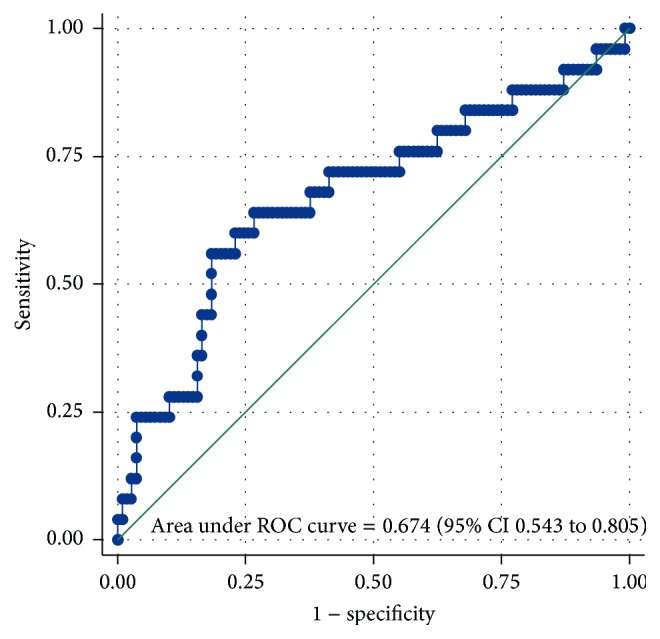
Receiver operating characteristic curve.

**Figure 3:**
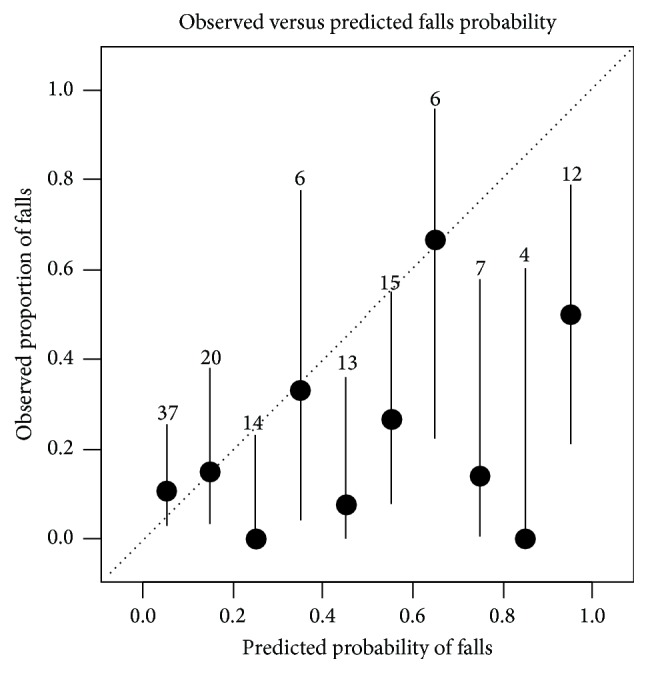
Probability for fall. Calibration plot of observed versus estimated probability for fall.

After calculation of the predicted probability to fall based on the values from our questionnaire and the coefficients published in the meta-analysis (Table 2), the prediction model yielded an AUC value for the discrimination of 0.67 (96% CI 0.54 to 0.81) (Figure 2). There was statistically significant miscalibration (*p* value from the Hosmer-Lemeshow test <0.00001) (Figure 3).

### 3.5. Understandability of the Questionnaires

Ten participants stated that some questions were unclear and they provided seven specific comments, such as the following: that they were diagnosed with hypertension but had normal blood pressure under medication and did not know what to answer in the questions about present diseases; that some questions were asking about two different pieces of information and that some questions had double negations.

## 4. Discussion 

In this longitudinal cohort study with a six-month follow-up of falls, including 134 community-dwelling elderly participants aged 60 years or more, we tested a preliminary version of an online questionnaire to assess the fall-risk. The main findings were that (a) it is feasible to do an online survey of a comprehensive set of fall-risk factors and (b) the understandability of the questions was good with the exceptions of five questions, (c) the response rate of the monthly falls assessment was too low, (d) the discrimination was moderate, and (e) the calibration was insufficient.

The strength of our study was the approach to devise a set of questions covering the whole spectre of risk factors for falls based on published meta-analyses. This study is an important first step in the development of a comprehensive self-administered questionnaire. Although we cannot present a final version of the questionnaire, this study provides important information for the future development of fall-risk questionnaires.

There are some limitations of our project. The understandability was assessed by semistructured interviews with experts and with an open question in the online questionnaire for the participants. We interviewed only two laypersons before we sent out the questionnaire to the participants. Interviewing of more participants before sending the questionnaire to the participants might have eliminated some problems with the understandability. It is challenging to assess risk factors with self-administered questionnaires. The different visual risk factors especially such as distant contrast sensitivity or depth perception [61] or the dual task problems are difficult to assess. Furthermore, snowball-sampling is a “biased” sampling technique because it is not random and the inclusion of the next participants depends on the previous participants (i.e., participants are not independent). An alternative would have been to search participants by the means of flyers or newspaper or radio advertisements. However, the snowball-sampling has the advantage of being nonexpensive and fast. The nonrandomness is not a large disadvantage in a feasibility study. A further limitation is the low response rate for the monthly fall-risk assessment. We did not systematically send reminders if participants did not respond. Furthermore, we did not present a fall definition to the participant, because we thought that this could confuse more than it would help. Presenting and explaining a fall definition such as the one used by Tinetti et al., “a sudden, unintentional change in position causing an individual to land at a lower level, on an object, the floor, or the ground, other than as a consequence of sudden onset of paralysis, epileptic seizure, or overwhelming external force” [62], might clarify what to report as a fall. Furthermore, the questions about the falls could include examples to illustrate what we understand by a fall. For example, some participants do not consider falling on their knees as a fall, because they were not “lying” on the ground after the fall. It is unclear whether the inclusion of photographs or graphical illustration could improve the reporting. Questions for falls could include examples of specific situations. However, our falls incidence of 19% is compatible with one-year incidences (39% for women, 30% for men), data recently published from Germany [63]; therefore we do not believe that there is an underestimation of the falls. We only assessed falls during six months; a longer follow-up would have increased the number of falls. The frequency of problems reported in the individual fall-risk questions was very low if compared to other studies on self-report fall-risk questionnaires [15, 17]. This could be due to the good health state of our participants but it could also be due to how the questions were formulated (i.e., unclear wording or wording targets only serious problems). Given the very low proportion of present risk factors, the selection of our sample could be problematic. There might be a selection bias towards a higher socioeconomic state, given the high proportion of participants with higher education. Given the low presence of risk factors we would have expected a lower falls incidence rate. Our prediction formula still overestimates the fall-risk. This is most probably due to the high correlation between the included predictors. However, our sample size was sufficiently large for univariable analyses but too low to adjust for this correlation by the means of a multivariable model. Therefore, the prediction formula needs to be adjusted with methods proposed by Steyerberg and colleagues [51] in a larger sample once the questionnaire is in its definitive version and after testing of the reliability.

If we compare our results to published studies using questionnaires for the assessment of fall-risk, we have similar values for calibration and discrimination compared to Cattelani et al. [16]. Compared to El Miedany et al. [15] we have lower predictive values; they received an AUC value of 0.89 with only five predictors. However, they included a sample where all had at least one previous fall and where 82% reported to walk slower, 65% reported loss of balance, and 55% had poor sight. Therefore, the two samples are not similar. Our AUC value is low but one has to consider that other tests widely used to predict falls, such as the timed-up-and-go (TUG) test, do not have better predictive values. A recent review on the predictive values for falls of the TUG in community-dwelling elderly people found an AUC value of 0.57 [64].

We did not find an association between self-perceived fall-risk and falls. One might expect that the self-perceived risk for falling increases fear of falls, which is known to be associated with future falls. One reason why we did not find an association is that the response options of the question for the self-perceived risk were not optimal and should be improved for future studies.

Our study has some implications for further research. The following amendments need to be done before further testing: (1) the question about past falls which should ask about the number of falls in the last year; it is recommended that persons with more than one fall in the past year should be referred to a detailed assessment [65]; (2) rewording of some questions; and (3) explication of what is considered as a fall to exclude falls, for example, due to an overwhelming external force, that is, following the falls definition used by [62]. After a refinement of the questions, test-retest reliability must be tested before the coefficients for a final predictive model should be assessed with a multivariable logistic regression based on results from a larger cohort study with a one-year follow-up in which the analyses should be separated for the prediction of one fall or recurrent falls. Furthermore, a larger sample size would allow evaluating whether some questions might be eliminated without losing discrimination or calibration of the prediction tool.

Implications for practice are as follows. Our study showed that in a sample with a relative low risk profile the incidence of falls was 19% during a period of six months and that the participants were mostly not aware of their fall-risk. Health professionals who see patients for other indications, for example, for the treatment of osteoarthritis, back pain, or neurological problems, could use this fall-risk questionnaire as a screening tool or a “flag system” and specifically test the domains where the patients report problems. The health professionals could then refer the patient to a falls-prevention group. The tool could also be used for the preparation of a visit to a medical doctor. The patients could bring the questionnaire to the medical doctor to discuss the results and possible strategies if necessary.

## 5. Conclusion 

This study showed that fall-risk awareness is low and that even in a sample of elderly people with a low risk profile in known risk factors the falls incidence is 19% in a six-month period. The present questionnaire needs some adaptation of the wording and reliability testing before a definitive prediction formula can be developed in a large sample and with multivariable analyses. Measures need to be implemented to increase the monthly response rate for the follow-up period.

## Figures and Tables

**Table 1:** Comparison of existing falls prediction tools for community-dwelling older adults.

Name of tool	Publication	Fall outcome	Risk factors used in the final model	Follow-up period (months)	Area under the ROC-curve (if not stated otherwise)	Number of participants	Person with falls *N* (%)	Number of falls
AGS/BGS/AAOS algorithm	AGS/BGS/AAOS [18] Tested in [19]	Any fall	*Self-reported and performance measures* How many falls have you had in the last year? Balance/gait impairment	12	Likelihood ratio+ of 2.71, likelihood ratio− of 0.61	117	52 (44.4%)	

Geriatric Postal Screening Survey (GPSS)	Alessi et al., 2003 [20]	Any fall	*Self-reported* 10 items: falls, balance problems, urinary incontinence, depression, memory loss, functional impairment, health perceptions (two questions), weight loss, polypharmacy, pain	12	Sensitivity 0.94, specificity 0.51	147		

“Sturz-Risiko-check” (Falls-risk check)	Anders et al., 2006 [17]	Any Fall	*Self-reported* Questionnaires covering: vision problems, polymedication, depressive mood, stopped riding bicycle, neurological disease, balance disorder, weight loss, fear of falls, sit-to-stand, heart diseases, slower in activities of daily living, falls experience, injuries as consequences of falls	Cross-sectional study (test of reliability)		117		
	Bongue et al., 2011 [21]	Any fall	*Self-reported and performance measure* Gender, living alone, psychoactive drug use, osteoarthritis, previous falls, change in the position of the arms during the one-leg balance	12	0.70 (0.67–0.73)	1795	563 (32%)	
	Buatois et al., 2010 [22]		*Self-reported and performance measure* History of falls, living alone, medications, female, five-times-sit-to-stand test	25		619		

FRAT-up	Cattelani et al., 2015 [23]	Any fall	*Self-reported and performance measure* Age, cognition impairment, depression, comorbidity, dizziness and vertigo, fear of falling, female sex, gait problems, hearing impairment, history of falls, history of stroke, instrumental disability, living alone, number of medications, pain, Parkinson, physical activity limitation, physical disability, poor self-perceived health status, rheumatic disease, urinary incontinence, use of antiepileptics, use of antihypertensives, use of sedatives, vision impairment, walking aid use	36	0.64 (0.61–0.67)	977	215 (22%)	
	Covinsky et al., 2001 [24]	Retrospective; fall or falls in the previous year	*Self-reported and performance measure* Abnormal mobility, balance difficulties or dizziness, fall in previous year	60	0.71		95 (33%)	197

EFST	Cwikel et al., 1998 [25]		*Self-reported and performance measure* Near falls, fall in the past year, injurious fall in the past year, walking speed, examiner impression of gait					

DFRA	Demura et al., 2011 [26]	Any fall	*Self-reported and performance measure* 50 items representing the five factors “symptoms of falling,” “physical function,” “disease and physical symptoms,” “environment,” and “behaviour and character”	Retrospective falls history	85.4% correct classified	1122	177 (16%)	

FRAS	El Miedany et al., 2011 [15]	Any Fall	*Self-reported and performance measure* Age, history of any fall, history of more than 1 fall, slowing of walking speed/change in gait, loss of balance, weak hand grip, poor sight	Case-control	0.89 (0.86–0.92)	985	559 (57%)	
	Gadkaree et al., 2015 [27]	Any fall	Age, gender, race, self-reported balance problem, history of fall	12	0.70 (0.67–0.78)	2539		
	Hirase et al., 2014 [28]		*Self-reported* **Have you fallen during the past year? Can you cross the street without resting (during a green traffic signal)? Can you continue to walk for an entire kilometer? Can you put on socks while standing on one leg?** Can you wring out a wet towel? **Have you admitted yourself to a hospital within the past year?** Do you feel dizzy upon standing up? **Have you ever had a stroke?** Have you ever been diagnosed with diabetes? Do you take sleeping pills, antihypertensive drugs, or minor tranquillizers? Do you often wear sandals or slippers? Can you see the letters in a newspaper, or a person's face, clearly? Can you hear a person's voice during a conversation? Do you often stumble or slip in your own house? **Do you have a fear of falling or do you hesitate to go out because you have a fear of falling?** *(Bold = the seven questions used for the prospective study)*	3	0.73 (0.62–0.83) for the seven bold factors; 0.82 (0.70–0.95) for all 15 factors	292	45 (15.6%)	

Modified Johns Hopkins fall-risk assessment tool mJH-FRAT	Hnizdo et al., 2013 [29]	Any fall	*Self-reported and performance measure* Age categories, fall history, elimination problems, high-risk medications, use of patient care equipment, limited mobility, altered cognition	During home health services (2 to 80 days)	0.66 (0.55–0.78)	107	33 (31%)	

Physiological profile assessment	Lord et al., 2003 [30]		*Self-reported and performance measure* Visual acuity-high contrast, yisual acuity-low contrast, edge contrast sensitivity, visual field dependence, proprioception, tactile sensitivity-ankle, vibration sense-knee, ankle dorsiflexion force, knee extension force, knee flexion force, reaction time-hand, reaction time-foot, sway on floor-eyes open, sway on floor-eyes closed, sway on foam rubber mat-eyes open, sway on foam rubber mat-eyes closed					
	Palumbo et al., 2015 [31]		*Self-reported and performance measure* Number of previous falls, number of drugs, self-perceived health status, previous falls (yes/no), drugs for dementia (yes/no), CESD depressed mood scale, if you are retired, do you have a veteran pension?, can you walk 300 meters twice without stopping?, gait speed, antihypertensive medication, do you have difficulty walking 400 meters on rough terrain?, antidepressants, walking posture: cautious attitude?, sibling with diabetes?, must you hold onto something (e.g., bannister) while climbing stairs?, quinolone antibacterials, antihypertensives	36	0.64 (0.61–0.67)	976		0.42 falls per person-year

LASA fall-risk profile	Peeters et al., 2010 [32]	At least 2 falls within 6 months	*Self-reported and performance measure* How often did you fall during the past 12 months, including the last fall?, do you often have dizzy spells?, are you able to use your own method of transport or public transportation?, are you able to go up 15 steps without standing still?, are you able to cut your own toenails?, grip strength of right hand, grip strength of left hand, body weight, do you have a dog or a cat?, how concerned are you that you might fall when … (10 activities listed)?, do you sometimes drink alcohol?, what is the highest level of education that you completed with a certificate?	12	0.65 (0.58–0.72)	408	76 (recurrent fallers, 18.6%)	
	Pluijm et al., 2006 [33]	Prospective, recurrent falling at 3-year follow-up	*Self-reported and performance measure* ≥2 falls in the previous year, dizziness, functional limitations, grip strength (men ≤ 56 kg, women ≤ 32 kg), body weight (women ≤ 62 kg; men ≤ 70 kg), fear of falling, dogs or cats in household, education ≥ 11 year, alcohol use (≥18 consumptions per week), alcohol use × education, ≥2 falls in the previous year × fear of falling	36	0.71 (0.67–0.74)	1214	55.3%	2570

Fall-risk assessment and screening tool FRAST	Renfro and Fehrer, 2011 [14]		*Self-reported and performance measure* 15 items: age, gender, fall history, daily physical activity level, number of prescription medicines, eye care, glasses or contacts, getting dizzy, use of assistive devices to walk, self-perceived risk behaviour, social activity, home-safety checklist, modified falls efficacy scale, mood scale, timed-up-and-go test.					

FRQ	Rubenstein et al., 2011 [13]		*Self-reported* I have fallen in the last 6 months. I am worried about falling. Sometimes, I feel unsteady when I am walking. I steady myself by holding onto furniture when walking at home. I use or have been advised to use a cane or walker to get around safely. I need to push with my hands to stand up from a chair. I have some trouble stepping up onto a curb. I often have to rush to the toilet. I have lost some feeling in my feet. I take medicine that sometimes makes me feel light-headed or more tired than usual. I take medicine to help me sleep or improve my mood. I often feel sad or depressed. Because I do not see well, I have difficulty avoiding hazards in my path, such as tree roots or electrical cords. (This last item was dropped due to low kappa with clinical evaluation.)					

FROP-COM	Russell et al., 2008 [34]		13 risk factors covered with 26 questions. Number of falls in the past 12 months, walking safely in the house, observation of balance, incontinence, number of medical conditions, vision deficit, assistance required to perform personal ADLs, number of fall-risk medications, assistance required to perform domestic ADLs, somatosensory deficit, cognitive status, level of physical activity, foot problems, number of medications, food intake, weight loss, nocturia, alcohol intake, inappropriate footwear, injury in past 12 months	12	0.68 (0.63–0.74)	344	164 (47.6%)	

FROP-COM screen	Russell et al., 2009 [35]	Any fall	*Self-reported and performance measure* Number of falls in the past 12 months, observation of balance, assistance required to perform domestic ADLs	12	0.73 (0.67–0.79)	344	164	
	Stalenhoef et al., 2002 [36]	Prospective, recurrent falls (≥2 falls)	*Self-reported and performance measure* Women, age (≥80), ≥2 falls in previous year, depression, SCL90 ≥ 22; hand dynamometry (men ≤ 22 kg, women ≤ 12 kg), postural sway abnormal	36	0.79	287		

Thai-FRAT	Jittima Thamarpirat et al., 2008 [37]	Any fall	*Self-reported and performance measure* Six factors: history of falls, impaired body balance, female, specific medication use, impaired visual acuity, Thai style house	24	Positive Likelihood-Ratio: 2.34	115		

QuickScreen clinical fall-risk assessment	Tiedemann et al., 2010 [38]	Multiple falls	*Self-reported and performance measure* Falls in past year, total medications, psychoactive meds, visual acuity test (MAR), touch sensation test, alternate step test, sit-to-stand test, tandem stand test	12	0.72 (0.66–0.79)	362	179 (49%)	
	Tromp et al., 2001 [39]	Any fall	*Self-reported and performance measure* Previous falls, urinary incontinence, visual impairment, use of benzodiazepines	12	0.65	1280	33%	

Empty cells indicate that we did not found any corresponding information in the publications.

**Table 2:** Predictors. Predictors found in review of reviews and odds ratios from an existing self-administered fall-risk questionnaire (multivariable odds ratios) and our questionnaire (univariable odds ratio).

Variable	*N* studies	*N* patients	Odds ratio	(95% CI)	Log of the odds ratio	Heterogeneity (*I* ^2^)	Meta-analysis	OR El Miedany (FRAS)	OR Obrist
Age (per 5-year increase)	8		1.11	(1.05–1.17)	0.10	*p* = 0.007	Deandrea et al., 2010 [5]	1.02 (1.01 to 1.04)	1.69 (1.14 to 2.53)
*Living alone*	12	5419	1.16	(1.02–1.32)	0.15	*I* ^2^ < 25%	Bloch et al., 2010 [40]		
Taking drugs (yes/no)	5	508	4.24	(3.06–5.88)	1.45	16%	Bloch et al., 2013 [4]		2.68 (1.02 to 7.02)
*Laxatives*	7	7 611	2.03	(1.52–2.72)	0.71	0%	Bloch et al., 2013 [4]		
*Psychotropic drugs*	32	43 552	1.74	(1.56–1.95)	0.55	0%	Bloch et al., 2013 [4]		
Polymedication	29	25 098	1.71	(1.50–1.96)	0.54	38%	Bloch et al., 2013 [4]		3.04 (1.18 to 7.89)
*Benzodiazepines*	26	54 919	1.61	(1.35–1.93)	0.48	0%	Bloch et al., 2013 [4]		
Antidepressants	46	19 988	1.59	(1.43–1.75)	0.46	21%	Bloch et al., 2013 [4]		
*Antiepileptics*	9	34 021	1.56	(1.28–1.90)	0.45	22%	Bloch et al., 2013 [4]		
*Antiparkinsonians*	13	21 899	1.55	(1.21–1.97)	0.44	42%	Bloch et al., 2013 [4]		
*Hypnotics*	36	4 453	1.53	(1.40–1.68)	0.43	44%	Bloch et al., 2013 [4]		
*Digoxins*	11	8 587	1.48	(1.11–1.99)	0.39	17%	Bloch et al., 2013 [4]		
*Narcotics*	7	22 973	1.43	(1.27–1.61)	0.36	39%	Bloch et al., 2013 [4]		
*Tranquilizers*	12	12 391	1.42	(1.21–1.67)	0.35	47%	Bloch et al., 2013 [4]		
Metabolic and endocrine medicines	11	38 846	1.39	(1.20–1.62)	0.33	0%	Bloch et al., 2013 [4]		
Antipsychotics	23	29 584	1.37	(1.16–1.61)	0.32	15%	Bloch et al., 2013 [4]		
Analgesics	11	9 869	1.33	(1.07–1.65)	0.29	0%	Bloch et al., 2013 [4]		
Anti-inflammatory	18	22 209	1.25	(1.11–1.42)	0.22	31%	Bloch et al., 2013 [4]		
ACE inhibitor	6	66 696	1.21	(1.15–1.28)	0.19	39%	Bloch et al., 2013 [4]		
Vasodilators	17	7 212	1.12	(1.04–1.21)	0.11	71%	Bloch et al., 2013 [4]		
Antihypertensives	25	81 908	1.1	(1.05–1.16)	0.10	25%	Bloch et al., 2013 [4]		
*Cardiovascular medicines*	23	88 467	0.78	(0.67–0.90)	−0.25	80%	Bloch et al., 2013 [4]		
Fall history	39	25 808	3.86	(3.42–4.37)	1.35	58%	Bloch et al., 2013 [4]	1.5 (0.8 to 2.7)	2.1 (0.78 to 5.69)
Fear of falling	13	22 809	2.4	(2.07–2.77)	0.88	48%	Bloch et al., 2013 [4]		1.35 (0.37 to 4.99)
*Digestive disease*	8	10 649	2.2	(1.65–2.93)	0.79	0%	Bloch et al., 2013 [4]		
Sensory disorder	9	3 125	2.2	(1.56–3.11)	0.79	38%	Bloch et al., 2013 [4]		1.54 (0.48 to 5.03)
Parkinson's disease	29	39 477	2.19	(1.68–2.84)	0.78	50%	Bloch et al., 2013 [4]		
Neurological disease	17	20 281	2.18	(1.69–2.82)	0.78	0%	Bloch et al., 2013 [4]		2.28 (0 to 11.45)
*Disorientation*	8	8 648	2.16	(1.68–2.78)	0.77	0%	Bloch et al., 2013 [4]		
Low body mass index	12	15 396	2.05	(1.70–2.48)	0.72	0%	Bloch et al., 2013 [4]		4.5 (0.27 to 73.83)
*Impaired cognition/dementia*	35	59 363	1.96	(1.80–2.14)	0.67	88%	Bloch et al., 2013 [4]		
Fracture history	11	1612	1.89	(1.53–2.34)	0.64	51%	Bloch et al., 2013 [4]		Not estimable
Urinary incontinence	34	59 458	1.73	(1.60–1.88)	0.55	49%	Bloch et al., 2013 [4]		2.23 (0 to 17.89)
*Self-perceived state problem*	18	19 015	1.73	(1.48–2.02)	0.55	56%	Bloch et al., 2013 [4]		
Depression	39	67 858	1.64	(1.52–1.76)	0.50	40%	Bloch et al., 2013 [4]		2.38 (0 to 19.27)
*Urinary functional sign*	4	1 826	1.64	(1.16–2.33)	0.50	19%	Bloch et al., 2013 [4]		
*Closely vision impairment*	7	13 418	1.62	(1.48–1.78)	0.48	0%	Bloch et al., 2013 [4]		
Cardiac and vascular problems	14	24 367	1.6	(1.45–1.75)	0.47	0%	Bloch et al., 2013 [4]		1.98 (0.82 to 4.76)
Vision impairment	39	38 671	1.49	(1.39–1.59)	0.40	47%	Bloch et al., 2013 [4]	2.8 (1.1 to 7.6)	3.07 (0.58 to 16.43)
*Anemia*	5	502	1.47	(1.15–1.88)	0.39	0%	Bloch et al., 2013 [4]		
*Stroke*	49	54 336	1.44	(1.34–1.56)	0.37	20%	Bloch et al., 2013 [4]		
*Cardiac rhythm disorder*	9	3 402	1.42	(1.14–1.75)	0.35	0%	Bloch et al., 2013 [4]		
Hearing impairment	17	21 878	1.37	(1.27–1.48)	0.32	11%	Bloch et al., 2013 [4]		1.3 (0.53 to 3.18)
*Ophthalmic disease*	16	31 443	1.29	(1.18–1.40)	0.26	24%	Bloch et al., 2013 [4]		
*Altered general health state*	9	13 786	1.28	(1.15–1.43)	0.25	34%	Bloch et al., 2013 [4]		
High blood pressure	35	45 115	1.28	(1.19–1.37)	0.25	42%	Bloch et al., 2013 [4]		2.09 (0.81 to 5.43)
Diabetes	40	61 028	1.27	(1.19–1.36)	0.24	11%	Bloch et al., 2013 [4]		1.47 (0 to 10.9)
*Behavioral disorder*	16	35 858	1.27	(1.14–1.42)	0.24	0.25	Bloch et al., 2013 [4]		
Postural hypotension	20	11 939	1.27	(1.09–1.47)	0.24	9%	Bloch et al., 2013 [4]		1.18 (0.44 to 3.22)
Arthrosis/osteoarthritis	37	5 284	1.24	(1.20–1.28)	0.22	88%	Bloch et al., 2013 [4]		0.76 (0.3 to 1.94)
*Low cognitive score*	22	1 754	1.22	(1.12–1.32)	0.20	46%	Bloch et al., 2013 [4]		
*Cancer*	10	26 642	1.22	(1.09–1.35)	0.20	44%	Bloch et al., 2013 [4]		
Abnormal walking test	11	14 677	3.34	(2.36–4.72)	1.21	0%	Bloch et al., 2013 [4]		
*Wandering*	3	8 896	2.82	(2.30–3.46)	1.04	0%	Bloch et al., 2013 [4]		
Muscular weakness	11	12 705	2.66	(2.12–3.33)	0.98	66%	Bloch et al., 2013 [4]	2.1 (1.1 to 4.4)	4.5 (0.27 to 74.51)
Slow walking speed	8	3 358	2.56	(1.96–3.33)	0.94	0%	Bloch et al., 2013 [4]	5.0 (2.0 to 12.0)	Not estimable
Walking problem	29	20 309	2.48	(2.24–2.75)	0.91	78%	Bloch et al., 2013 [4]		
Unable to get up from a chair	10	9 973	2.44	(1.92–3.09)	0.89	78%	Bloch et al., 2013 [4]		
*Unsteady turning*	6	1 274	2.39	(1.50–3.79)	0.87	77%	Bloch et al., 2013 [4]		
Self-perceived balance problem	4	3 118	2.36	(1.83–3.03)	0.86	28%	Bloch et al., 2013 [4]	3.5 (1.5 to 7.5)	1.84 (0.66 to 5.17)
*Abnormal Tinetti test score*	9	3 749	2.35	(1.63–3.40)	0.85	77%	Bloch et al., 2013 [4]		
*Abnormal balance test*	16	7 814	2.26	(1.79–2.85)	0.82	78%	Bloch et al., 2013 [4]		
*Reduction of step length*	9	1565	2.12	(1.46–3.08)	0.75	76%	Bloch et al., 2013 [4]		
*Balance problem*	14	4 318	2.1	(1.72–2.55)	0.74	90%	Bloch et al., 2013 [4]		
*Lower extremity disability*	19	8 691	1.89	(1.65–2.17)	0.64	78%	Bloch et al., 2013 [4]		
*Lower grip strength*	7	5 923	1.78	(1.44–2.21)	0.58	47%	Bloch et al., 2013 [4]		
Reduced mobility	11	24 526	1.58	(1.44–1.74)	0.46	0%	Bloch et al., 2013 [4]		1.18 (0.47 to 2.97)
*Feet problems*	18	15 014	1.5	(1.23–1.82)	0.41	54%	Bloch et al., 2013 [4]		
Dizziness	20	22 142	1.36	(1.13–1.63)	0.31	12%	Bloch et al., 2013 [4]		1.71 (0.69 to 4.24)
Limited activity	13	16 198	1.32	(1.01–1.72)	0.28	0%	Bloch et al., 2013 [4]		
*Physical activity*	26	8 981	0.79	(0.71–0.88)	−0.24	50%	Bloch et al., 2013 [4]		
Walking aid	36	54 336	2.1	(1.90–2.32)	0.74	61%	Bloch et al., 2013 [4]		9.39 (1.17 to 0)
*Nonadapted shoes*	5	1 671	1.97	(1.48–2.62)	0.68	83%	Bloch et al., 2013 [4]		
Obstacle on the ground	6	7 522	1.73	(1.55–1.93)	0.55	24%	Bloch et al., 2013 [4]		1.54 (0.48 to 5.03)
Disturbance of one or more ADL	13	17206	2.28	(2.10–2.48)	0.82	58%	Bloch et al., 2010 [40]		Not estimable
Frequent toileting	1	533	1.92	(1.12–3.27)	0.62	—	Sherrington et al., 2010 [41]		1.26 (0.47 to 3.34)
Pain	14	17926	1.56	(1.36–1.79	0.44	52%	Stubbs et al., 2014 [42]		1.61 (0.63 to 4.08)
Dual task	21		1.33	(1.18–1.50)	0.29	89%	Chu et al., 2013 [43]		Not estimable

Empty cells indicate that the values were not reported in the meta-analyses. Italic indicates variables that were not assessed with our questionnaire.

**Table 3:** Distribution of risk factors. Distribution of risk factors for all participants, for the nonfallers, and for the fallers. All responses are self-reported values and not observed or measured values.

Variables	All		Non fallers during 6 months of follow-up		Fallers during 6 months of follow-up		Risk ratio (95% CI)
	*N* responders	*N* (%) if not stated otherwise	*N* responders	*N* (%) if not stated otherwise	*N* responders	*N* (%) if not stated otherwise	
Age	134	69.28 (5.59)		68.65 (5.4)		72 (5.66)	
Body mass index	134	25.95 (4.04)		25.85 (4)		26.4 (4.25)	
Women	134	60 (45%)	109	49 (45%)	25	11 (44%)	0.97 (0.47 to 1.98)
Obese persons (BMI ≥ 30)	134	18 (13%)	109	15 (14%)	25	3 (12%)	0.88 (0.29 to 2.64)
BMI < 18.5	134	2 (1%)	109	1 (1%)	25	1 (4%)	2.75 (0.66 to 11.52)
Fallen during last 12 months	134	2 (1%)	109	1 (1%)	25	1 (4%)	2.75 (0.66 to 11.52)
Emergency room visit after fall in last 12 months	134	24 (18%)	109	17 (16%)	25	7 (28%)	1.78 (0.84 to 3.79)
Fracture after fall during last 12 months	134	2 (1%)	109	1 (1%)	25	1 (4%)	2.75 (0.66 to 11.52)
Commotio after fall during last 12 months	134	1 (1%)	109	0 (0%)	25	1 (4%)	5.54 (3.86 to 7.96)
Fear of falls after fall during last 12 months	134	1 (1%)	109	0 (0%)	25	1 (4%)	5.54 (3.86 to 7.96)
Resting more than half an hour on floor after fall in last 12 months	134	4 (3%)	109	2 (2%)	25	2 (8%)	2.83 (0.99 to 8.06)
Needing help for activities of daily living	134	0 (0%)	109	0 (0%)	25	0 (0%)	0 (0 to 0)
Using walking aids	134	1 (1%)	109	0 (0%)	25	1 (4%)	5.54 (3.86 to 7.96)
Walking slower or making more breaks	134	3 (2%)	109	1 (1%)	25	2 (8%)	3.8 (1.57 to 9.17)
Problems crossing road at traffic light during green phase	134	1 (1%)	109	1 (1%)	25	0 (0%)	0 (0 to 0)
Stumbling at least once per months	134	0 (0%)	109	0 (0%)	25	0 (0%)	0 (0 to 0)
Unsecure if walking in darkness	134	2 (1%)	109	0 (0%)	25	2 (8%)	5.74 (3.96 to 8.32)
Unsecure even if walking in daylight	134	3 (2%)	109	1 (1%)	25	2 (8%)	3.8 (1.57 to 9.17)
Vision problems	134	1 (1%)	109	0 (0%)	25	1 (4%)	5.54 (3.86 to 7.96)
Seeing good without glasses	134	5 (4%)	109	3 (3%)	25	2 (8%)	2.24 (0.72 to 6.98)
Seeing good with glasses	134	25 (19%)	109	24 (22%)	25	1 (4%)	0.18 (0.03 to 1.28)
Does not see good with glasses	134	106 (79%)	109	84 (77%)	25	22 (88%)	1.94 (0.62 to 6.01)
Hearing problem	134	4 (3%)	109	2 (2%)	25	2 (8%)	2.83 (0.99 to 8.06)
Hearing problem but wearing hearing aid	134	42 (31%)	109	33 (30%)	25	9 (36%)	1.23 (0.59 to 2.56)
Taking sedative	134	5 (4%)	109	4 (4%)	25	1 (4%)	1.08 (0.18 to 6.44)
Taking blood pressure decreasing medication	134	2 (1%)	109	0 (0%)	25	2 (8%)	5.74 (3.96 to 8.32)
Taking antiepileptics	134	47 (35%)	109	34 (31%)	25	13 (52%)	2.01 (1 to 4.04)
Taking antidepressants	134	2 (1%)	109	2 (2%)	25	0 (0%)	0 (0 to 0)
Other medicaments	134	3 (2%)	109	1 (1%)	25	2 (8%)	3.8 (1.57 to 9.17)
More than one medicament	134	51 (38%)	109	39 (36%)	25	12 (48%)	1.5 (0.74 to 3.03)
Taking at least one medicament	134	26 (19%)	109	17 (16%)	25	9 (36%)	2.34 (1.17 to 4.68)
Low spirit at more than half of all days	134	3 (2%)	109	1 (1%)	25	2 (8%)	3.8 (1.57 to 9.17)
Low spirit at some days	134	51 (38%)	109	39 (36%)	25	12 (48%)	1.5 (0.74 to 3.03)
No disease	134	26 (19%)	109	17 (16%)	25	9 (36%)	2.34 (1.17 to 4.68)
Parkinson	134	78 (58%)	109	59 (54%)	25	19 (76%)	2.27 (0.97 to 5.33)
Stroke	118	3 (3%)	97	2 (2%)	21	1 (5%)	1.92 (0.37 to 9.97)
Epilepsy	134	37 (28%)	109	27 (25%)	25	10 (40%)	1.75 (0.86 to 3.54)
Diabetes	134	40 (30%)	109	35 (32%)	25	5 (20%)	0.59 (0.24 to 1.46)
Incontinence	134	1 (1%)	109	0 (0%)	25	1 (4%)	5.54 (3.86 to 7.96)
Osteoarthritis	134	4 (3%)	109	3 (3%)	25	1 (4%)	1.35 (0.24 to 7.68)
Rheumatic disease (including osteoarthritis)	134	2 (1%)	109	2 (2%)	25	0 (0%)	0 (0 to 0)
Multiple sclerosis	134	4 (3%)	109	3 (3%)	25	1 (4%)	1.35 (0.24 to 7.68)
Neurologic disease	134	3 (2%)	109	2 (2%)	25	1 (4%)	1.82 (0.35 to 9.39)
High blood pressure	134	39 (29%)	109	32 (29%)	25	7 (28%)	0.95 (0.43 to 2.09)
Fainting	134	44 (33%)	109	37 (34%)	25	7 (28%)	0.8 (0.36 to 1.76)
Leg pain during walking	134	0 (0%)	109	0 (0%)	25	0 (0%)	0 (0 to 0)
Dyspnea	134	6 (5%)	109	4 (4%)	25	2 (8%)	1.86 (0.56 to 6.1)
Heart symptoms	134	28 (21%)	109	20 (18%)	25	8 (32%)	1.78 (0.86 to 3.7)
Dizziness	134	29 (22%)	109	23 (21%)	25	6 (24%)	1.14 (0.5 to 2.6)
Wanted weight loss	118	54 (46%)	97	43 (44%)	21	11 (52%)	1.3 (0.6 to 2.83)
Unwanted weight loss	116	31 (27%)	97	24 (25%)	19	7 (37%)	1.6 (0.69 to 3.69)
Difficulties getting up from a chair	134	42 (31%)	109	31 (28%)	25	11 (44%)	1.72 (0.85 to 3.47)
Self-perceived balance problem	134	36 (27%)	109	27 (25%)	25	9 (36%)	1.53 (0.74 to 3.15)
Back pain	134	36 (27%)	109	27 (25%)	25	9 (36%)	1.53 (0.74 to 3.15)
Hip pain	129	9 (7%)	106	7 (7%)	23	2 (9%)	1.27 (0.35 to 4.58)
Knee pain	134	4 (3%)	109	2 (2%)	25	2 (8%)	2.83 (0.99 to 8.06)
Foot pain	134	3 (2%)	109	1 (1%)	25	2 (8%)	3.8 (1.57 to 9.17)
Pain yes/no	134	22 (16%)	109	16 (15%)	25	6 (24%)	1.61 (0.73 to 3.56)
Limited ROM hip	134	22 (16%)	109	16 (15%)	25	6 (24%)	1.61 (0.73 to 3.56)
Limited ROM knee	134	44 (33%)	109	35 (32%)	25	9 (36%)	1.15 (0.55 to 2.39)
Limited ROM ankle	134	20 (15%)	109	18 (17%)	25	2 (8%)	0.5 (0.13 to 1.94)
Any ROM limitation	134	37 (28%)	109	31 (28%)	25	6 (24%)	0.83 (0.36 to 1.91)
Need to get up during night	134	37 (28%)	109	31 (28%)	25	6 (24%)	0.83 (0.36 to 1.91)
Sensory deficit in lower extremity	134	21 (16%)	109	17 (16%)	25	4 (16%)	1.02 (0.39 to 2.68)
Fear of falls	134	85 (63%)	109	67 (62%)	25	18 (72%)	1.48 (0.67 to 3.3)
Low standing confidence	134	16 (12%)	109	12 (11%)	25	4 (16%)	1.4 (0.55 to 3.57)
Perceived dual task problems	134	24 (18%)	109	20 (18%)	25	4 (16%)	0.87 (0.33 to 2.31)
Cables on the floor at home	134	5 (4%)	109	5 (5%)	25	0 (0%)	0 (0 to 0)
Loose carpets at home	134	39 (29%)	109	31 (28%)	25	8 (32%)	1.15 (0.54 to 2.43)
Objects on the floor	134	97 (72%)	109	78 (72%)	25	19 (76%)	1.21 (0.52 to 2.79)
Objects on the stairs	134	16 (12%)	109	12 (11%)	25	4 (16%)	1.4 (0.55 to 3.57)
Thresholds in doors	134	13 (10%)	109	10 (9%)	25	3 (12%)	1.27 (0.44 to 3.67)
No bath mat	134	22 (16%)	109	16 (15%)	25	6 (24%)	1.61 (0.73 to 3.56)
Any home hazard	134	1 (1%)	109	0 (0%)	25	1 (4%)	5.54 (3.86 to 7.96)
Weak legs	134	24 (18%)	109	19 (17%)	25	5 (20%)	1.15 (0.48 to 2.75)
